# Household Exposures to Polybrominated Diphenyl Ethers (PBDEs) in a Wisconsin Cohort

**DOI:** 10.1289/ehp.0900839

**Published:** 2009-08-04

**Authors:** Pamela Imm, Lynda Knobeloch, Carol Buelow, Henry A. Anderson

**Affiliations:** 1 Wisconsin Department of Health Services, Madison, Wisconsin, USA and; 2 Wisconsin State Laboratory of Hygiene, Madison, Wisconsin, USA

**Keywords:** bromine, passive air sample, PBDE, vacuum dust, X-ray fluorescence

## Abstract

**Background:**

Human exposure to polybrominated diphenyl ethers (PBDEs) is virtually universal in the United States. Although the uses of these chemicals as flame retardants in fabrics, foams, and plastics are well defined, human exposure pathways are not well understood.

**Objectives:**

This study was designed to assess current PBDE body burdens and identify residential sources of exposure among 29 men and 15 women in 38 households.

**Methods:**

Portable X-ray fluorescence (XRF) analyzers were used to measure bromine levels in upholstered furnishings, bedding, vehicle interiors, and electronic devices. Vacuum cleaner contents, indoor air samples, and blood sera were analyzed for PBDE congeners using conventional gas chromatograph methods.

**Results:**

Bromine levels varied widely within similar household items. The greatest range for upholstered items was found among vehicle seat cushions (7–30,600 ppm). For electronic devices, television sets ranged from 4 ppm to 128,300 ppm. Based on mixed effects modeling, adjusting for couple households, the bromine content in the participants’ sleeping pillows and primary vehicle seat cushions were the strongest predictors of log lipid-adjusted blood serum PBDE concentrations (*p*-values = 0.005 and 0.03, respectively). The total pentaBDE congener levels found in dust samples and in passive air samples were not significant predictors of blood sera levels.

**Conclusions:**

This study demonstrates the usefulness of the portable XRF analyzer in identifying household items that may contribute to human exposure to PBDEs.

Polybrominated diphenyl ethers (PBDEs) have been used as chemical flame retardants since the late 1970s [Agency for Toxic Substances and Disease Registry (ATSDR) 2004]. Commercial formulations include pentaBDE, which is found in polyurethane foams and used in household furnishings, vehicle upholstery, and bedding; octaBDE, which is used in plastics for personal computers and small appliances; and decaBDE, which is incorporated into plastics for television cabinets, automotive interiors, aircraft, consumer electronics, wire insulation, and back coatings for upholstery (ATSDR 2002). The technical formulations for pentaBDE and octaBDE were withdrawn from the U.S. marketplace in 2004. Previously manufactured consumer products containing these formulations will remain in use and will continue to be exposure reservoirs. For example, decaBDE continues to be used in plastic coverings for televisions, computers, and other electronic devices [(U.S. Environmental Protection Agency (EPA) 2006].

Global monitoring programs have found traces of PBDEs in fish, birds, and mammals. Exposure is virtually universal in the United States, and serum levels are approximately 10-fold higher in North American residents than in Europeans and Asians (Hites 2004; Schecter et al. 2005). In U.S. studies, investigators have documented serum levels ranging more than 2 orders of magnitude. This finding suggests that dietary and environmental factors may be important determinants of exposure (Anderson et al. 2008. Potential sources of exposure include indoor air, house dust, direct contact with treated products, and contaminated foods. Several investigators have identified PBDEs in meat, fish, and dairy products (Domingo 2004; Domingo et al. 2006; Harrad et al. 2004; Manchester-Neesvig et al. 2001; Schecter et al. 2006). In an analysis of dietary exposures, Schecter et al. (2008) concluded that although fish tend to be more highly contaminated (median, 616 pg/g) than meat (median, 190 pg/g) and dairy products (median, 32.2 pg/g), meat is the most important food source in the United States. Wu et al. (2007) found significant associations between PBDE levels in human breast milk and the consumption of meat and dairy products. Lorber (2008) evaluated exposure pathways for food and water ingestion, inhalation, and ingestion and dermal contact with household dust and concluded that food was a minor source of exposure; whereas house dust accounted for 82% of human intake estimates in the United States. Lorber (2008) also noted that intake estimates ranged from 3 ng/day to 400 ng/day, with the large range partially the result of different intake-rate assumptions.

Animal studies have shown that PBDEs can affect liver function and alter hormone levels (Bloom et al. 2008; Dunnick and Nyska 2009; Meeker et al. 2009; Turyk et al. 2008). PBDEs may also be toxic to the developing nervous system (Costa and Giordano 2007). Viberg et al. (2003) found that a single dose administered to pregnant mice during a critical period of fetal development can cause permanent changes to behavior in pups. In June 2008, the U.S. EPA set safe daily exposure levels of 0.1, 0.1, 0.2, and 7 μg/kg body weight per day for BDE congeners 47, 99, 153, and 209, respectively (U.S. EPA 2008).

Several investigators have measured PBDE levels in household dust samples and calculated potential exposure rates. However, most household studies have not included biomonitoring. Thus, it is difficult to make direct comparisons between PBDE levels in dust and biological samples (Harrad et al. 2004; Stapleton et al. 2005; Wilford et al. 2005). Wu et al. (2007) found a positive association between PBDE levels in household dust and breast milk (*R* = 0.76).

PBDEs have also been measured in indoor air. Wilford et al. (2004) sampled air in 74 homes in Ottawa, Canada, during the winter of 2002–2003 using polyurethane foam passive air samplers (PUF). The BDE levels in the homes ranged across 3 orders of magnitude (from 2 to 3,600 pg/m^3^) and averaged 260 pg/m^3^. The major congeners were BDE-47 and BDE-99; both are major components of the pentaBDE formulation. Based on the median levels found in these homes, the authors concluded that the maximum daily exposure via the inhalation pathway would be 1.9–2.0 ng/day or about 4% of overall daily intake. Allen et al. (2007) used personal air samplers to assess exposure among 20 Boston area residents and concluded that indoor air might account for up to 11% of the non-209 BDE and 22% of BDE-209 exposure in U.S. adults.

In a novel approach, Allen et al. (2008b) validated the use of portable X-ray fluorescence (XRF) analyzers for measuring bromine concentrations in household items. These investigators demonstrated that XRF readings were highly correlated with gas chromatograph-mass spectrometer (GC-MS)–measured bromine and PBDE levels in furnishings and electronic devices (*R* = 0.93). By using XRF readings to identify bromine and confirming the presence of PBDEs by chemical analysis, these researchers demonstrated a clear relationship between the presence of brominated flame retardant–treated products in the home and pentaBDE congener levels in household dust.

Analysis of serum samples from 508 members of a cohort of Great Lakes residents found that sport-fish ingestion rates were strongly predictive of polychlorinated biphenyl (PCB) and dichlorodiphenyldichloroethylene (DDE) serum levels but weakly associated with PBDE levels (Anderson et al. 2008). To identify household sources of PBDE exposure, we designed a follow-up study to evaluate residential exposure routes such as indoor air, household dust, and exposure to bromine-containing household items. Using methods described by Allen et al. (2008b), we used a portable XRF to measure bromine content in upholstered furnishings, vehicle interiors, televisions, and computers. We also collected venous blood samples, vacuum dust, and indoor air samples for analysis.

## Materials and Methods

### Human subjects

The protocol for this study was approved by the University of Wisconsin–Madison Human Subjects Institutional Review Board. All subjects signed informed consent forms before participating.

### Subject recruitment

A total of 29 men and 15 women were recruited from an existing cohort of 3,692 Great Lakes frequent and infrequent consumers of sport fish that was established in 1993. The construction of this original cohort, which included residents of five Great Lakes states, has been described elsewhere (Hanrahan et al. 1999). Letters of invitation were mailed to all 110 Wisconsin cohort residents who had been tested for PCB, DDE, and PBDE serum levels in 2004–2005. Of these 110 residents, 44 agreed to participate.

### Study design

Participants were scheduled for a home visit and sent questionnaires that requested information on demographics and diet; the age of their house; the type of heating system used; hours of television and computer use; details about the participant’s primary vehicle and hours of use; use of a boat; hobbies related to crafts with plastic, foam, or fabric; and work environment. During the home visits, which were conducted in April and May 2008, the questionnaires were reviewed for completeness, a blood sample was drawn, and bromine readings were taken of upholstered furniture, computers, televisions, and the driver’s seat cushion of the participant’s primary vehicle using a Niton X-ray fluorometer (XRF). In addition, vacuum contents were collected and a PUF passive air sampler was placed in the most used room of the house other than the bedroom. The PUF remained in the participant’s home for approximately 30 days before being returned for analysis. PUFs, serum samples, and vacuum contents were sent to the Wisconsin State Laboratory of Hygiene (WSLH) in Madison, Wisconsin, for analysis.

### XRF readings

During the home assessment, bromine readings were taken of upholstered furniture (including mattresses, mattress pads, and pillows), appliances (televisions and computers only), and the driver-side seat of the participant’s primary vehicle using a Niton XRF analyzer. The XRF provides a nondestructive estimate of the bromine content that correlates closely with laboratory analyses (Allen et al. 2008b). The technology illuminates a sample with high-energy photons, dislodging electrons and resulting in the release of fluorescent X-ray patterns unique to each element. The XRF calculates a concentration by measuring the scattered X-rays. Pretesting determined that 30-sec readings provided reproducible results with confidence intervals generally ≤ 2% of the reading. For bromine, the XRF has a limit of detection (LOD) of 4 ppm. The reading was taken on the front casing of electronic appliances. For upholstered items, readings were taken from the center of the seat cushions.

### Blood serum

For the PBDE analyses, whole blood (15–20 mL) was drawn into red-top, glass Vacutainer tubes, allowed to clot for 20 min, and centrifuged for 15 min. Serum was transferred to hexane-rinsed glass vials and stored at −20°C. Thawed samples were extracted with hexane/ethyl ether. Cleanup and fractionation were attained using Florisil, silica gel, and concentrated sulfuric acid. The concentrated sample was injected onto an Agilent GC-MS with negative ion chemical ionization and selective ion monitoring (Agilent Technologies, Santa Clara, CA). We analyzed 24 congeners (BDE 17, 28, 47, 49, 66, 71, 77, 85, 99, 100, 119, 126, 138, 153, 154, 156, 183, 184, 191, 196, 197, 206, 207, and 209), and quantified seven (BDE congeners 28, 47, 85, 99, 100, 153, and 154) on a 30-m DB-5HT capillary column. BDE-154 was found to coelute with polybrominated biphenyl (PBB-153) and is not included in total serum PBDE levels in this analysis (Anderson et al. 2008). Quality control was monitored using method blanks, spikes of bovine serum, and sample duplicates. Method blanks consisted of solvents and reagents carried through the entire extraction, cleanup, and analysis procedure. Sample data were flagged if the concentration was not more than twice that found in the method blank. This was done for one very low result for BDE-47 and for three very low results for BDE-99. For the seven PBDE congeners detected, the mean of five bovine serum spike recoveries ranged from 87% for BDE-28 to 95% for BDE-47. Only BDE-47 was reportable in three duplicate samples. For these duplicates, the relative percentage difference ranged from 4.5% to 23%. Standard reference materials from the National Institute of Standards and Technology (NIST; Gaithersburg, MD) were tested to confirm analytical techniques in serum. Results for BDE congeners 47, 99, 100, and 153 (average of five analyses) ranged from 101% (BDE-47 and BDE-99) to 111% (BDE-153) of the NIST values.

Another vial of blood was drawn for lipid analyses: 3 mL of whole blood was collected in a plastic vial, allowed to clot, and then centrifuged for 15 min. Serum was analyzed for total, nonfasting, cholesterol and triglyceride levels. These levels were analyzed to adjust PBDE serum levels for total serum lipids according to the following formula (Phillips et al. 1989):


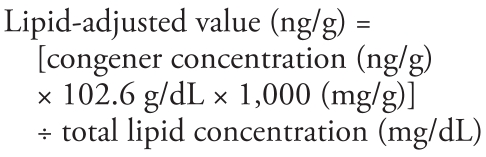


Total serum lipids were calculated as


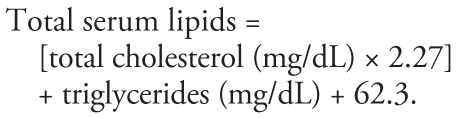


### Household vacuum dust

During the home assessment, interviewers collected vacuum bags or emptied dust and dirt from bagless vacuums into hexane-rinsed glass jars. Although the length of time the bag had been in use was not known, these samples were likely to represent recently collected dust from the household. This method was chosen for convenience and cost-effectiveness. Its major weakness is the inability to attribute the sample to any particular area for any specific period of time. At the time the home visit was scheduled, we asked participants not to empty their vacuum contents in order to have enough mass to test for PBDEs. We generally collected bags at least half full or were able to fill the glass jars with the bagless contents.

We tested 38 dust samples for BDE congeners 47, 99, 100, 153, 154, 206, 207, and 209. The WSLH limited the dust analysis to the expected major PBDE components. Dust samples were passed through a #18 sieve (1-mm opening). This size sieve was chosen to provide a homogeneous sample without inclusion of coarse particles. We extracted 1 g of dust particles using pressurized fluid extraction (Dionex model ASE 200; Dionex Corporation, Sunnyvale, CA) with dichloromethane. Cleanup and fractionation were attained using Florisil, silica gel, and concentrated sulfuric acid. The extract was concentrated to 2.0 mL, and internal standard was added. The concentrated sample was injected onto a dual-column gas chromatograph equipped with electron-capture detectors. The primary column was a 60 m × 0.25 mm × 0.1 μm DB-1 column, with confirmation on a 60-m DB-5 column. Quality control was monitored by use of method blanks, lab matrix spikes, duplicates, surrogate spikes, and confirmation of the analytes by second column or GC/MS. Confirmation was based on retention time agreement and reasonable agreement of the analytical results. PBDEs were not detected in the method blanks. Spikes were added to a finely powdered diatomaceous earth. Average recoveries for seven spikes ranged from 84% for BDE-47 to 104% for BDE-209. Surrogate spike recoveries for BDE-119, which was added to every sample, averaged 83%. The average percentage difference between duplicate samples (*n* = 12) ranged from 5.2% for BDE-47 to 31% for BDE-209. Standard reference materials from NIST were tested to confirm analytical techniques. Results for seven of the eight congeners tested ranged from 75% (BDE-154) to 113% (BDE-209) of the NIST value. NIST did not report a result for BDE-207.

### Passive air sample

PUF disks mounted in Tisch passive air sampler housings (Tisch Environmental Inc., Cleves, OH) were used to assess exposure to vapor-phase PBDEs. Measurements of air volume were not taken; therefore, air concentrations could not be calculated. PUFs were placed at eye level or above to avoid accidental contact or displacement. We placed these away from air vents, doors, and windows. Before placement, PUFs, which measured 14 cm diameter × 1.35 cm thickness, were cleaned using Soxhlet extraction followed by sonication before being deployed in homes. The PUF disks were placed in the participant’s most used room other than a bedroom because we expected these nonbedroom areas to include a wider variety of upholstered furnishings and electronic devices. We allowed participants to choose the location (most often the living room or family room and frequently on top of the television set in these rooms). After approximately 30 days, participants placed the PUF in a hexane-rinsed glass jar, completed a lab slip indicating the date, and mailed the PUF, lab slip, and Tisch housing to WSLH. Thirty-eight PUFs were sent to the WSLH and analyzed for 23 congeners (the same as those analyzed in blood serum, less BDE-119).

The exposed disk was Soxhlet extracted in hexane for 16 hr. The extract was cleaned using silica gel and concentrated sulfuric acid and concentrated to 1.0 mL. An internal standard was added, and the extract was analyzed for PBDEs by GC/MS with negative ion chemical ionization and selected ion monitoring. The GC column was a 30-m DB-5HT. Quality control was monitored using method blanks, PUF blanks, field blanks, lab matrix spikes for all congeners, and surrogate spikes. Blanks were run on PUF disks before they were deployed. The report limit for BDE-47 was set at the average plus two times the standard deviation of the level in the blanks. Method blanks were clean, and results were not blank subtracted. Of the three field blanks, only one contained BDE-47 and BDE-99 in amounts exceeding the LOD, which we did not consider significant relative to the amount found in the samples. Average recoveries of spikes to clean PUF disks (*n* = 5) ranged from 72% (BDE-17) to 99% (BDE-209). Surrogate spike recoveries for BDE-119, added to every sample, averaged 99%.

### Statistical methods

Analyses were conducted using SAS statistical software (version 9.1; SAS Institute Inc., Cary, NC). Because of the nonnormal distribution of serum, we log transformed the total lipid-adjusted serum levels. We also conducted nonparametric correlation statistics, regression analyses, and mixed effects modeling. In this article, statistical significance is defined as a *p*-value ≤ 0.05.

For congener-specific PBDE values for serum, dust, and PUF samples, we calculated the geometric means. For samples with any specific congener value < LOD for that sample and congener (Table 1). For sum PBDEs, congener-specific PBDE values for serum, dust, and PUF samples were summed to yield a total for each participant. To calculate the sum, values below the LOD for any specific congener were imputed as 0. This was done in order to not overinflate levels by the summation of several imputed congener-specific values where levels were originally below the LOD.

## Results

### Demographics

A total of 29 men and 15 women who resided in 38 Wisconsin households participated in this study. Their mean age was 58 years (range, 43–77). All participants reported their race as white. Annual incomes, provided by 36 households, exceeded $55,000/year in 22 households (61%), ranged from $35,000 to $55,000/year in 10 households (28%), and were < $35,000/year in 4 households (11%).

### Comparisons of serum levels from 2004–2005 and 2008

**Figure 1:**
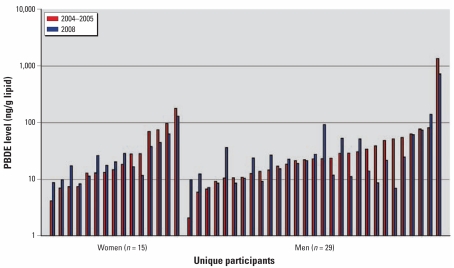
Log-transformed, lipid-adjusted PBDE levels for 2004–2005 and 2008 among women and men.

Log-transformed, lipid-adjusted serum PBDE levels measured in 2008 were closely correlated with 2004–2005 levels and did not change significantly over this time period (Figure 1). Geometric mean concentrations decreased < 2%. Among women, geometric mean levels increased from 21.08 to 21.60 ng/g, providing an average increase of < 3%. The two measurements among women were highly correlated (*p* < 0.0001). Men experienced, on average, a decrease of < 4% (mean levels decreased from 24.06 to 23.19 ng/g). The separate measurements among men were also highly correlated (*p* < 0.0001).

### PBDE detection in blood, vacuum dust, and PUFs

Detection frequencies for PBDEs in vacuum cleaner dust, PUF, and 2008 blood serum samples are shown in Table 1. The predominant congener found in blood was BDE-47; it contributed 51–100% of the body burden in our subjects (mean, 85%). BDE-99 was detected in more than half of our volunteers, whereas BDE-100 and BDE-153 were less common. The congeners found in serum also dominated the residues in the passive air samples, where BDE-47 and BDE-99 comprised 68% and 14% of the total residues, respectively. BDE-209, the major residue present in vacuum dust, was detected in only two passive air samples and was not found in any of the serum samples. BDE congeners 154, 206, and 207 were also found only in the vacuum dust samples.

### Household exposure to PBDEs

As shown in Table 2, bromine levels in household items and automobile interiors varied widely. For example, television bromine readings ranged from 4 ppm to 128,300 ppm. Although levels tended to be lower in newly manufactured liquid crystal display (LCD) sets, some older cathode ray (CRT) models had similarly low bromine levels. Bromine levels in mattresses and mattress pads ranged from below detection to 14,600 ppm. Bed pillows varied according to their filling material, with polyurethane foam pillows tending to be high in bromine and polyester fiber and feather pillows containing almost no bromine.

### Comparison of blood PBDE levels with vacuum dust, PUFs, and XRF measurements

We used mixed effects analysis with adjustment for couple households (there were 6 two-participant households and 32 single-participant households) to identify predictors of PBDE levels in serum. Based on our model, with one male outlier removed, the bromine levels of the participant’s sleeping pillow and the vehicle seat cushion were significant predictors of log lipid-adjusted blood serum levels (Table 3; *p* = 0.005 and 0.027, respectively). We categorized the bromine levels into low, medium low, medium high, and high for pillows (≤ 1,500, > 1,500 to ≤ 7,500, > 7,500 to ≤ 13,500, and > 13,500 ppm) and into low, medium, and high for vehicle seat cushions (≤ 3,000, > 3,000 to ≤ 21,000, and > 21,000 ppm). We constructed these categories based on histogram distributions of the XRF bromine readings. We initially included total pentaBDE congener levels (congeners 47, 99, 100, 153, and 154) in PUF and dust samples in the model, but these were not significant. We analyzed the total pentaBDE congener levels in these samples as a separate grouping and compared them with sum serum levels because these congeners are more commonly found in serum. We also included XRF bromine measurements of the household appliances (family room television and household computer) and bedding in the initial model, but these were not significant predictors of serum PBDE levels. The lack of significance of bromine readings from appliances in predicting PBDE levels in serum is most likely due to high levels of BDE-209 in electronics, which we did not detect in any of the blood samples.

Mixed effects analysis based on the sum of XRF readings from upholstered furniture in the participant’s living room (couch and chair) and bedroom (top layer of bedding and sleeping pillow) was also predictive of log lipid-adjusted serum PBDE levels (*p* = 0.0097). We categorized the cumulative bromine levels into low, medium, and high groups based on histogram distribution data (< 30,000, 30,000–105,000, and > 105,000 ppm). However, the sum of XRF readings from electronic devices (family room television and computer) was not predictive.

### Predictors of BDEs in PUF and vacuum dust samples

We compared PBDE levels in dust and PUF samples to assess correlations between airborne particulates and dust that collects on vacuumed surfaces within a home. Spearman’s nonparametric test revealed no correlation. We also considered air movement as a potential factor in PUF and dust PBDE levels. We conducted nonparametric Kruskal-Wallis tests for significant differences between furnace type (a dichotomous variable: forced air or “other”) and the total PBDE levels in dust, and separately for the total PBDE levels in PUFs, and found no associations. Age of house (built pre-1970built pre-1970–1990, and post-1990) was not correlated with PBDE levels in the PUFs or vacuum dust samples. We considered these tests “household-level” tests and used 38 households for analysis (data not shown).

We conducted additional analyses of total pentaBDE congener levels (congeners 47, 99, 100, 153, and 154) and of BDE-209 only in dust compared with XRF readings for the family/living room sofa, family/living room upholstered chair, computer, family room television, top layer of bedding (mattress or mattress pad), and sleeping pillow. These were household items and household dust samples; therefore, we analyzed 38 households, averaging pillow readings for couple households. We found no correlations with these dust levels (total pentaBDE congener levels or BDE-209 levels) and the bromine levels for any of these household items. We compared the analysis of total pentaBDE congener levels in PUFs with XRF readings of living room furniture only (the PUF was most often placed in the living room). Spearman’s correlation tests revealed that the XRF readings for the television set and the upholstered living room chair were significantly correlated with total pentaBDE congener levels in PUFs (*p* = 0.03 and 0.04, respectively). BDE-209 levels in PUFs were not associated with living room XRF readings.

### Other variables

We analyzed other survey variables but do not present them here because of their lack of significant correlation with PBDE blood levels. These variables included dietary information on fish and dairy consumption, motorboat use, and employment and hobby information.

## Discussion

We designed this study to assess current PBDE body burdens and identify residential sources of exposure among a subsample of adults who provided blood samples for PBDE analysis in 2004–2005 (Anderson et al. 2008). Comparison of exposure risk factors from our initial multistate study of 508 volunteers showed that those with PBDE levels in the upper 90th percentile were more likely to be men, were older, had lower household incomes, spent more time outdoors, had higher PCB and DDE body burdens, reported more years of sport-fish consumption, and were more likely to sleep on a waterbed than were those in the lowest 10th percentile. Most of these findings were consistent with findings for other bioaccumulative toxins, but the waterbed finding was unique and suggested that one’s choice of mattress and, perhaps, other household furnishings might be important determinants of exposure to brominated flame retardants. This study was designed to investigate this hypothesis.

Lipid-adjusted PBDE levels in this cohort did not change significantly over the 4-year follow-up period. The geometric mean value of 23.0 ng/g from our 2004–2005 study of this group was similar to the summed geometric means for BDE-47 (20.7 ng/g) and BDE-153 (6.0 ng/g) in the 2003–2004 National Health and Nutrition Examination Survey (NHANES) survey (Sjodin et al. 2008).

As was previously reported by Allen et al. (2008b), XRF readings varied widely among similar household items. For example, bromine levels were much higher in fabric car seats than in leather upholstered seats (mean, 7,140 vs 290 ppm) and were lower in newer LCD televisions than in older CRT sets. Levels in mattresses and mattress pads ranged from below detection in an innerspring mattress to 6,710 ppm in a waterbed. Allen et al. (2008b) found a range of < 5 to 2,481 ppm among their sample of mattresses, although we do not know whether waterbeds were measured or which types of mattresses had the highest levels of detection. XRF readings from polyurethane foam pillows used by 18 study participants averaged 3,646 ppm, whereas feather and polyester fiber pillows had much lower levels (mean, 6 and 107 ppm, respectively). Allen et al. (2008b) reported a bed pillow bromine range of < 5 to 3,512 ppm but did not distinguish types of pillows.

Laboratory analyses found wide ranges in PBDE levels in passive air samplers as well. Total PBDE levels in the PUFs ranged across 2 orders of magnitude, from 3 to 461 ng/PUF. Few studies have used PUFs for exposure assessment. Wilford et al. (2004) converted the concentration in the PUF to picograms per cubic meter by applying a sampling rate of 2.5 m^3^/day. Using the same conversion, our concentrations ranged from 43.5 to 5117.8 pg/m^3^ (mean, 562.1 pg/m^3^), considerably higher than those reported by Wilford et al. (2–3,600 pg/m^3^). Wilford et al. (2004), however, did not detect BDE-49 or BDE-209, which we detected in some of our samples (Table 1). These authors may have focused on the congeners likely to volatilize from the pentaBDE technical product; this would explain why BDE-209 was not detected. A comparison of BDE-47 alone also revealed considerably higher levels in our study: a range of 34.7–2555.6 pg/m^3^ among our samples compared with less than detection to 1,600 pg/m^3^ among samples reported by Wilford et al. (2004).

Total BDE levels in dust samples from these Wisconsin homes ranged from 0.47 to 46.88 ppm (mean, 5.65 ppm). These levels, which were measured in the spring of 2008, are similar to the dust levels reported by other North American researchers. In their 2004 study of PBDEs in house dust samples collected from 16 homes in Washington, DC, and one home in Charleston, South Carolina, Stapleton et al. (2005) found an average total concentration of 5,900 ng/g dry weight (5.9 ppm). Wilford et al. (2005) reported a mean PBDE concentration of 5.5 ppm in vacuum dust in 68 homes in Ottawa, Canada. However, another researcher collected carpet vacuum samples from nine Texas homes and found a higher average PBDE level of 12.1 ppm (Schecter et al. 2005). Allen et al. (2008a) compared BDE levels in home vacuum bags to levels in researcher-collected vacuum samples in Boston homes. They found significantly lower BDE levels in the dust from home vacuum bags, which had a geometric mean of 4.3 ppm, compared with bedroom and living room researcher-collected vacuum samples, which had geometric mean levels of 6.3 and 13.7 ppm. Analysis of vacuum bag contents may not be as effective a method for analysis of dust association with current blood serum levels because the exact time and location of dust collection are unknown.

The variables most strongly associated with log lipid-adjusted serum PBDE levels were the bromine measurements of pillows and the driver-side seat of the participant’s primary vehicle. These findings suggest that brominated flame retardants in pillows and automotive seats are released by off-gassing or breakdown of polyurethane foam and fabrics, resulting in human exposure. Because it is assumed that most individuals spend many hours per day in close proximity to their pillow and bedding, it is not surprising that when PBDEs are present in these they would be a major contributor to exposure. Although we analyzed specific household items and their associations with higher serum levels in this study, cumulative bromine readings for living rooms and respondent bedrooms were tested in a mixed effects model and were significant predictors of blood serum levels (*p* = 0.0097). We categorized the total bromine level in households into low, medium, and high groups based on sleeping pillow, top layer of bedding, and living room couch and upholstered chair—the most common items found in each household.

This study demonstrates the usefulness of the XRF analyzer in identifying items that may contribute to human exposure to PBDEs. The range of XRF readings for similar-appearing items demonstrated that for dose estimation it is not sufficient to simply quantify the different furnishings in a home. Although the distribution of BDE congeners in PUFs was quite different from that in vacuum dust, the technical difficulty of collecting and analyzing PUF samples and their limited predictive contribution to our models made them the least useful of sampling methods employed in our study. PUFs required chemical cleaning before deployment in homes. Although PBDE levels in vacuum dust samples were not correlated with blood levels among this study population, a more controlled vacuum sampling method has proven useful in other studies (Wu et al. 2007).

## Figures and Tables

**Table 1:** Congener levels detected in dust, PUF, and blood serum.

	Geometric mean, % detected (LOD)		
	Dust	PUF	Lipid-adjusted serum
PBDE congener	(*n* = 38, ng/g)	(*n* = 38, ng/PUF)	(*n* = 44, ng/g)
BDE-17	NA	1.35, 61% (0.5)	ND (0.025)
BDE-28	NA	2.03, 95% (0.5)	2.01, 5% (0.025)
BDE-47	520, 100% (20)	15.17, 100% (0.7)	19.11, 100% (0.025)
BDE-49	NA	2.00, 45% (0.5)	ND (0.025)
BDE-66	NA	2.70, 34% (0.5)	ND (0.025)
BDE-85	NA	3.68, 5% (0.5)	1.95, 2% (0.025)
BDE-99	614, 100% (20)	2.79, 97% (0.5)	4.06, 55% (0.025)
BDE-100	120, 92% (20)	1.25, 66% (0.5)	2.77, 23% (0.025)
BDE-153	73, 84% (20)	3.55, 8% (0.5)	4.53, 11% (0.05)
BDE-154	51, 79% (20)	ND (0.5)	ND (0.05)
BDE-206	51, 61% (40)	ND (10)	ND (0.5)
BDE-207	32, 29% (40)	ND (10)	ND (0.5)
BDE-209	1,398, 100% (80)	5.48, 5% (10)	ND (0.5)
Sum of BDEs^a^	3,385, 100%	22.40, 100%	22.65, 100%

Abbreviations: NA, not analyzed; ND, not detected.

a Dust sum of BDEs are congeners 47, 99, 100, 153, 154, 206, 207, and 209; PUF sum BDEs are congeners 17, 28, 47, 49, 66, 85, 99, 100, 153, and 209; serum sum BDEs are congeners 28, 47, 85, 99, 100, and 153. For individual congeners, values < LOD were imputed as half the LOD (except where no samples were found to have a specific congener). Sums were based on adding all congeners; nondetected values were imputed as 0 for these sums.

**Table 2:** XRF bromine readings in household items and vehicles and PBDE levels in household dust and passive air filters.

Tested item	No.	Range (mean)
XRF bromine readings (ppm)		
Primary vehicle seat	44	7–30,600 (5,463)
Cloth upholstery	32	7–30,600 (7,139)
Leather	11	20–2,669 (288)
Other	1	8,769
All household televisions	98	4–128,300 (94,338)
CRT before 2005	73	56,000–128,300 (92,330)
CRT after 2005	11	93,300–108,300 (100,880)
LCD before 2005	2	101,400–113,800 (107,600)
LCD after 2005	12	4–106,800 (42,190)
Mattresses	36	No detect–6,707 (339)
Innerspring	32	No detect–2,282 (98)
Foam	1	8
Waterbed	2	18–6,707 (3,362)
Air	1	1,983
Mattress pads	32	No detect–14,600 (1,416)
Sleeping pillows	44	No detect–16,400 (1,537)
Polyurethane foam	18	No detect–16,400 (3,646)
Feather	8	No detect–16 (6)
Polyester fiber	18	No detect–1,877 (107)
Living room carpet	38	No detect–718 (130)
Living room sofa	38	No detect–19,400 (2,599)
Office computer	30	No detect–109,000 (31,546)

PBDE levels		
Household dust (ng/g)	38	466–46,883 (5,651)
Passive air sample (ng/PUF)	38	3.3–460.6 (44.8)

Where *n* = 1, range and mean are equal.

**Table 3:** Independent predictors of log-transformed, lipid-adjusted PBDEs (ng/g lipid) (*n* = 43).

Dependent variable	Predictor variable	Parameter estimate	Standard error	*p*-Value
Log lipid-adjusted PBDEs	Intercept	1.3552	0.3075	< 0.0001
	Pillow	0.6868	0.1198	0.0046
	Car seat	0.5647	0.1650	0.0267
